# Clinical characterisation and phylogeny of respiratory syncytial virus infection in hospitalised children at Red Cross War Memorial Children’s Hospital, Cape Town

**DOI:** 10.1186/s12879-016-1572-5

**Published:** 2016-05-31

**Authors:** Regina Oladokun, Rudzani Muloiwa, Nei-yuan Hsiao, Ziyaad Valley-Omar, James Nuttall, Brian Eley

**Affiliations:** Paediatric Infectious Diseases Unit, Red Cross War Memorial Children’s Hospital, Cape Town, South Africa; Department of Paediatrics and Child Health, University of Cape Town, Cape Town, South Africa; Division of Medical Virology, University of Cape Town, Cape Town, South Africa; National Health Laboratory Service, Grootes Schuur Hospital, Cape Town, South Africa; Centre for Respiratory Diseases and Meningitis, Virology, National Institute for Communicable Diseases, Sandringham, Johannesburg, South Africa; Faculty of Health Sciences, Department of Clinical Laboratory Sciences Medical Virology, University of Cape Town, Cape Town, South Africa

**Keywords:** Respiratory syncytial virus, Nosocomial infection, Clinical characteristics, Genetic diversity

## Abstract

**Background:**

Respiratory syncytial virus (RSV) is a major cause of lower respiratory tract infection in young children in both the community and hospital setting.

**Methods:**

The clinical presentation, patient and phylogenetic characteristicsof laboratory-confirmed cases of RSV, as well as risk factors for nosocomial infectionat Red Cross War Memorial Children’s Hospital in Cape Town were analysed. A multiplex PCR assay that detects 7 respiratory viruses was used to identify RSV nucleic acid on respiratory specimens.

**Results:**

A total of 226 children were studied, ages ranging between 1 week and 92.5 months (median: 2.8 months, IQR: 1.3–6.3 months) and 51.8 % were males. The median duration of symptoms prior to diagnosis was 2 days (IQR: 1–4 days). Nosocomial infections wereidentified in 22 (9.7 %) children. There were pre-existing medical conditions in 113 (50.0 %) excluding HIV, most commonly prematurity (*n* = 58, 50.0 %) and congenital heart disease (*n* = 34, 29.3 %). The commonest presenting symptoms were cough (196, 86.7 %), difficulty in breathing (115, 50.9 %) and fever (91, 41.6 %).A case fatality rate of 0.9 % was recorded. RSV group A predominated (*n* = 181, 80.1 %) while group B accounted for only 45 (19.9 %) of the infections. The prevalent genotypes were NA1 (*n* = 127,70.1 %), ON1 (*n* = 45,24.9 %) and NA2 (*n* = 9,5.0 %) for group A while the only circulating RSV B genotype was BA4. There was no significant difference in the genotype distribution between the nosocomial and community-acquired RSV infections. Age ≥ 6 months was independently associated with nosocomial infection.

**Conclusions:**

A large percentage of children with RSV infection had pre-existing conditions. Approximately one tenth of the infections were nosocomial with age 6 months or older being a risk factor. Though both RSV groups co-circulated during the season, group A was predominant and included the novel ON1 genotype. Continued surveillance is necessary to identify prevalent and newly emerging genotypes ahead of vaccine development and efficacy studies.

## Background

Respiratory syncytial virus (RSV) is an important cause of bronchiolitis and pneumonia in infants and young children [1]. Globally, it is estimated that RSV causes over 30 million new acute lower respiratory tract infection (LRTI) episodes annually, resulting in more than 3.4 million hospital admissions and199,000 deaths in children younger than 5 years of age [1]. One-third of RSV-related deaths occur in the first year of life, with 96 % of these deaths occurring in low-resource countries [1]. Repeated infections may occur throughout life as immunity following the first RSV infectiondoes not protect against subsequent infections [2]. The infection is spread mainly by close or direct contact with infectious secretions or after touching contaminated surfaces [3]. Risk factors associated with RSV disease include prematurity, cardiopulmonary and immunosuppressive diseases [4]. Nosocomial outbreaks of RSV, which usually coincide with seasonal outbreaks, are a major hazard in paediatric wards, especially in those individuals with increased risk of RSV infection. Nosocomial outbreaks are associated with prolonged hospitalisation and increased mortality compared to community-acquired illness [5–7]. HIV-infected children also experience prolonged shedding of RSV [6].

RSV is classified into groups A and B based on reactions with monoclonal antibodies against the fusion (F) and attachment (G) glycoproteins [8]. Both groups co-circulate within the community and health care institutions and predominance of one over the other varies by year and geographic location [9, 10]. Many genotypes from each group have been described. New genotypes are continuing to emerge while others are no longer detectable [11, 12]. Although the predominant group and overall patterns of circulating genotypes are distinct for different communities [11],emerging RSV genotypes are also thought to show global spread [13]. Different genotypes may co-circulate within a season, with certain genotypes dominating before being replaced with other genotypes [14, 15]. There are conflicting reports on the association of different groups and genotypes with severity of illness [16–19]. Ongoing surveillance of the clinical and molecular epidemiology of RSV genotypesis important to characterise prevalent and emerging genotypes that may impact on vaccine development.

The aims of the study were to determine the proportion of RSV nosocomial infections among children testing positive for RSV, describe clinical characteristics of patients with the infection, phylogenetically classify the genotypes causing nosocomial and community-acquired infection, and determine risk factors associated with nosocomial infection in children with acute LRTI, hospitalised at Red Cross War Memorial Children’s Hospital (RCWMCH), Cape Town.

## Methods

### Study population

The study took place at RCWMCH a tertiary referral hospital affiliated to the University of Cape Town and caring for children up to the age of 13 years. A retrospective cross-sectional study was conducted on patients with confirmed LRTI and RSV who had been hospitalised between January and October 2012. These patients were identified from the Division of Medical Virology laboratory database. The time period of January to October 2012 was chosen because the laboratory database detected the first cases of RSV in January and the last cases in October 2012.

### Clinical data collection

Clinical information was extracted retrospectively from hospital records, including demographic data, mode of infection (community-acquired vs. nosocomial), underlying medical conditions, potential risk factors for nosocomial RSV infection and disease outcome. The information was recorded on a standardised data collection form and anonymously entered into an Excel spreadsheet.

### Case definitions

Nosocomial RSV infection was diagnosed in a child who developed new symptoms and signs of LRTI at least 5 days after hospital admission and in whom RSV was detected in respiratory secretions collected during the new respiratory infection [20, 21].

Community-acquired RSV infection was diagnosed in a child with an acute LRTI present at the time of hospital admission or occurring withinor occurring within less than 5 days of admission and in who RSV was detected in respiratory secretions.

Acute LRTI was as per the attending clinicians’ diagnosis. A high-dependency or high-care unit is where specialist nursing care and monitoring of seriously ill patients is provided with a higher level of care available than on general wards but less than is given to patients in intensive care [22].

HIV infection in children aged <18 months was diagnosed by HIV DNA PCR and confirmatory HIV RNA PCR, and in children ≥18 months of age by HIV Rapid test and confirmatory HIV enzyme-linked immunosorbant assay (ELISA). HIV exposed uninfected refers to infants that are born to HIV positive women but tested negative by HIV PCR.

### Chest radiograph classification

Chest radiographs of the patients taken within 24 h of diagnosis of acute LRTI due to RSV infection were obtained. Antero-posterior and lateral images were viewed in most instances. The radiological findings were categorised according to the World Health Organization’s standardised classification for interpreting chest radiograph findings for epidemiological studies: (1) primary end-point consolidation or pleural effusion i.e. the presence of end-point consolidation or pleural effusion that meets criteria for primary end-point, (2) other consolidation or infiltrates i.e. the presence of other (non-end –point) infiltrates, and (3) no consolidation, infiltrate or effusion i.e. the absence of end point consolidation, other infiltrate or pleural effusion [23]. The radiographs were reported independently by 2 of the investigators (RO and BE), without knowledge of the clinical information of each patient. A consensus was reached for any images in which there was a discrepancy.

### Laboratory diagnosis of RSV infection, co-infections and RSV phylogenetic analysis

A commercial multiplex PCR assay (Seeplex RV7, Seegene, Seoul, South Korea) was used to screen for 7 respiratory viruses (Influenza A, Influenza B, Metapneumovirus, RSV A/B, Rhinovirus A, Parainfluenza 1/2/3, Adenovirus) on nasopharyngeal aspirate or bronchoalveolar lavage specimens. Bacterial and viral co-pathogens were identified on blood, tracheal aspirate or urine specimens obtained at the discretion of the attending clinicians. Diagnostic assays were performed at the Medical Virology and Microbiology Laboratories at Groote Schuur Hospital National Health Laboratory Service. Viral complementary DNA (cDNA) specimens generated during the performance of the diagnostic assay for all viral isolates were stored at -80 °C. These stored specimens were used to characterise the relatedness of the RSV isolates.

Viral phylogeny was determined by partial sequencing of the RSV G envelope glycoprotein based on previously published methodology [24]. PCR amplicons were sequenced using BigDye terminator chemistry and sequences determined on an ABI 3130XL genetic analyzer (Applied Biosystems, Carlsbad, USA). The phylogenetic analysis was done by multiple RSV isolates sequence alignments using BioEdit 7.09 (Ibis Biosciences, Carlsbad USA). Construction of neighbour-joining phylogenetic trees was computed by using MEGA 4.1 (The Biodesign Institute, Tempe, USA). Sequence differences were visualised using highlighter nucleotide transition and transversion plots (www.hiv.lanl.gov).

### Analytical methods

Data were entered and analysed using SPSS 22. The clinical information was analysed using conventional parametric and non-parametric statistical methods. Demographic, clinical and laboratory variables were explored using univariate analysis. Variables associated with nosocomial infection were analyzed using a multivariable logistic regression model. The level of significance was set at *p* < 0.05.

## Results

**Fig. 1:**
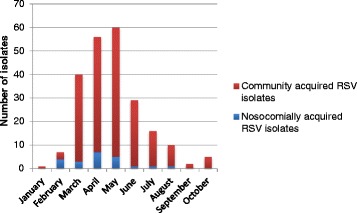
Monthly distribution of community-acquired and nosocomial acute lower respiratory tract infection associated with RSV

Two-hundred and twenty-six children with PCR-confirmed RSV acute lower respiratory tract infection were identified during the study period, January to October 2012. Figure 1 shows the monthly distribution of community-acquired and nosocomial acute respiratory tract infection associated with RSV. Case detection peaked in May.

### Characteristics of children with RSV infection

Table 1 shows the demographic characteristics and age distribution of the children with RSV infection. The median age of the children studied was 2.8 months (IQR: 1.3–6.3 months) and 160 (70.8 %) were less than 6 months of age. Pre-existing conditions other than HIV infection were present in 113 children (50.0 %). Table 2 shows the characteristics disaggregated according to different age groups. There were 117 males (51.8 %). A chest radiograph was obtained in 221 (97.8 %) of the cases and features consistent with lower respiratory tract infection were observed in 220 (97.4 %). HIV infection was confirmed in only 4 (1.8 %) children, 211 (83.4 %) were uninfected while the status of 11 (4.9 %) was unknown.

**Table 1:** Age and pre-existing conditions other than HIV infection

Parameter	*N* = 226
Median age (Months)	2.8
IQR	1.3–6.3
*Age group* (*months*)	
0 – <1	31 (13.7 %)
1- < 3	84 (37.2 %)
3- < 6	45 (19.9 %)
6 – <24	60 (26.5 %)
≥ 24	6 (2.7 %)
*Pre*-*existing conditions other than HIV infection*	*N* = 113 (50.0 %)
Prematurity	58 (51.3 %)
Congenital heart disease	34 (30.1 %)
Chronic lung disease	20 (17.7 %)
Previous IPPV	17 (15.0 %)
Trisomy 21	13 (11.5 %)
Previous cardiac surgery	7 (6.2 %)
^a^ Other conditions	7 (6.2 %)

^a^ Other conditions: Gastro-oesophageal Reflux Disease (*n* = 1), Guillain-Barré syndrome (*n* = 1), VATER (Vertebral defects, anal atresia, cardiac defects, tracheo-esophageal fistula and renal anomalies) syndrome (1), cerebral palsy (*n* = 2) and other chromosomal abnormalities (*n* = 2)

**Table 2:** Characteristics of children with RSV infection by age group

	*Age group* (*months*)					
Parameter	0 – <1	1– < 3	3– < 6	6 – < 24	≥24	Total
	*N* = 31(%)	*N* = 84(%)	*N* = 45(%)	*N* = 60(%)	*N* = 6(%)	*N* = 226(%)
*Gender*						
Male	17(54.8)	46 (54.8)	20 (17.1)	32 (53.3)	2(33.3)	117 (51.8)
Female	14 (45.2)	38 (45.2)	25 (55.6)	28 (46.7)	4 (66.7)	109 (48.2)
Chest X-ray conclusion						
Primary end-point consolidation or pleural effusion	9 (29.0)	27 (32.1)	7 (15.6)	11 (18.3)	2 (33.3)	56 (24.8)
Other consolidation/infiltrate	21 (67.7)	56 (66.7)	36 (80.0)	47 (78.3)	4 (66.7)	164 (72.6)
No consolidation/infiltrate/effusion	0 (0.0)	0 (0.0)	0 (0.0)	1 (1.7)	0 (0.0)	1 (0.4)
No X-ray	1 (3.2)	1 (1.2)	2 (4.4)	1 (1.7)	0 (0.0)	5 (2.2)
Weight-for-age Z- score						
> +2 (overweight)	0 (0.0)	1 (1.2)	0 (0.0)	1 (1.7)	0 (0.0)	2 (0.9)
+ 2 to -2 (appropriate)	22 (71.0)	54 (64.3)	21 (46.7)	33 (55.0)	4 (66.7)	134 (59.3)
< -2 to -3 (moderately underweight)	4 (12.9)	16 (19.0)	8 (17.8)	12 (20.0)	1 (16.7)	41 (18.1)
< -3 (severely underweight)	5 (16.1)	13 (15.1)	16 (35.6)	14 (23.3)	1 (16.7)	49 (21.7)
*HIV status*						
Unexposed uninfected	21 (67.7)	64 (76.2)	29 (64.4)	45 (76.7)	4 (66.7)	164 (72.6)
Exposed uninfected	8 (25.8)	18 (21.4)	14 (31.1)	7 (11.7)	0 (0.0)	47 (20.8)
Infected	1 (3.2)	0 (0.0)	1 (2.2)	1 (1.7)	1 (16.7)	4 (1.8)
Unknown	1 (3.2)	2 (2.4)	1 (2.2)	6 (10.0)	1 (16.7)	11 (4.8)
*Pre*-*existing conditions other than HIV infection*						
Yes	9 (29.0)	30 (35.7	29 (64.4	39 (65.0	6 (100.0)	113(50)
No	22 (71.0)	54 (64.3)	16 (35.6)	21 (35.0)	0 (0.0)	113(50)

### Clinical characteristics, management and outcome

The median duration of symptoms preceding hospitalisation was 2 days (IQR: 1–4 days). As shown in Table 3, the commonest presenting symptoms were cough 196 (86.7 %), difficulty in breathing (tight chest) 115 (50.9 %) and fever 91 (41.6 %). Wheezing was present in 20 (8.8 %) of the cases. With regards to the management of the patients, 170 (75.2 %) cases received supplemental oxygen, 89 (39.4 %) were admitted either in the intensive care or high care units while 59 (26.1 %) required assisted ventilation by continuous positive airway pressure (CPAP) or mechanical ventilation. Antibiotics were instituted in 221 (97.8 %), but confirmed bacterial infection was detected in only 24 (10.6 %) children. While 170 (75.2 %) were discharged, 54 (23.9 %) were transferred to a primary or secondary hospital for ongoing care. The median duration of stay in RCWMCH was 5.0 days (Range 1–90 days). Two patients died giving a mortality rate of 0.9 %.

**Table 3:** Clinical and laboratory characteristics, management and outcome of 226 patients with RSV infection

Parameter	Number of patients (%)
Presenting symptom	
Cough	196 (86.7)
Difficulty in breathing (tight chest)	115 (50.9)
Fever	91 (41.6)
Nasal congestion	67 (29.6)
Runny nose	35 (15.4)
Vomiting	26 (11.5)
Wheeze	20 (8.8)
Apnoea	12 (5.3)
Inflammatory and haematological markers	
Median CRP (mg/L)	12.3
Median WCC (x 10^9^/L)	11.1
Median percent band cell count	8.0
Median Haemoglobin concentration (g/dL)	11.0
Median platelet count (x 10^9^/L)	410
Treatment	
Supplemental oxygen	170 (75.2)
Bronchodilators	150 (66.4)
Assisted ventilation	*N* = 59 (26.1)
CPAP	43 (19.0)
IPPV	16 (7.1)
Antibiotics	*N* = 221 (97.8)
Intravenous	174 (78.7)
Oral	47 (21.3)
Presence of co-pathogens	70 (31.1)
Care level	*N* = 89 (39.4)
ICU	47 (20.8)
High care	38 (16.8)
Both ICU and high care	4 (1.8)
Outcome	
Discharged	170 (75.2)
Transferred to another hospital	54 (23.9)
Died	2 (0.9)

*CPAP* Continuous positive airway pressure, *IPPV* Intermittent Positive Pressure Ventilation, *ICU* Intensive care unit, *CRP* C-reactive protein, *WCC* White cell count

The first death occurred in an 8.8 month old male, who was HIV negative and hospitalised with community acquired RSV infection and acute gastroenteritis but no pre-existing condition. He also had adenovirus co-infection. He was nursed in intensive care unit (ICU) and required intermittent positive pressure ventilation (IPPV) but died as a consequence of respiratory failure on day 3 of hospitalisation. He had been infected with an RSV genotype BA4 isolate.

The second death occurred in a2.8 month old preterm female who was HIV negative andhad community acquired RSV infection. She was nursed in ICU and ventilated. She died on day 38 of admission from respiratory failure. She had been infected with a RSV ON1 genotype isolate.

### Co-pathogens during RSV-lower respiratory tract infection

Seventeen different viral, bacterial and fungal co-pathogens were identified in 70 (31.1 %) children. The commonest isolates were rhinovirus 22 (26.2 %), adenovirus 12 (14.3 %) and para-influenza virus 10 (11.9 %). Bacterial pathogens accounted for 24 (28.6 %) of the isolates, including 6 (7.1 %) isolated from blood cultures. The commonest bacterial isolates included *Staphylococcus aureus* 8 (9.5 %), *Escherichia coli* 2 (2.4 %) and *Pseudomonas aeruginosa* 2 (2.4 %).

### Genotype distribution and pattern of RSV infection

RSV A and RSV B accounted for 181 (80.1 %) and 45 (19.9 %) of the infections, respectively. There were no mixed infections with both A and B groups. The prevalent genotypes were NA1 (*n* = 127, 70.1 %), ON1 (*n* = 45, 24.9 %) and NA2 (*n* = 9, 5.0 %) for groupA, while the only circulating RSV B genotype was BA4. Age, gender, need for assisted ventilation, HIV status and presence of co-pathogens were not associated with the RSV genotypes.

**Fig. 2:**
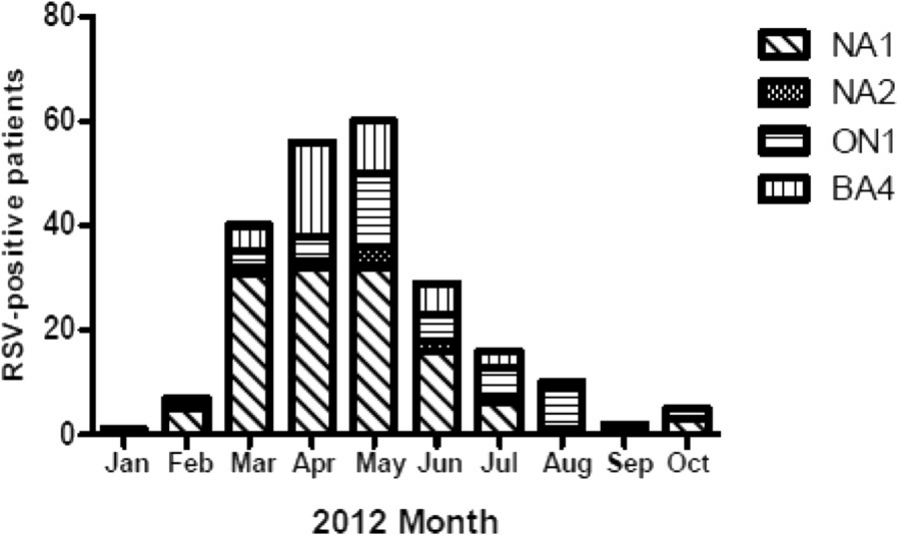
RSV genotypes prevalent in sampled cases of lower respiratory tract infection, 2012

The NA1 genotype predominated especially between the months of March to June (Fig. 2), and was the predominant genotype implicated in both community-acquired and nosocomial infections. There was no significant difference in the genotype distribution between the nosocomial and community-acquired RSV infections (Table 4).

**Table 4:** RSV genotypes in relation to nosocomial infection

RSV genotype	Nosocomial infection	Community acquired	Total	*P* value
	*n* = 22	*n* = 204	*N* = 226 (% of total)	
NA1	14 (63.6 %)	113 (55.4 %)	127 (56.2)	0.61
NA2	0 (0.0 %)	9 (4.4 %)	9 (4.0)	
ON1	5 (22.7 %)	40 (19.6 %)	45 (19.9)	
BA4	3 (13.6 %)	42 (20.6 %)	45 (19.9)	

### Characteristics of patients requiring assisted ventilation

In univariate analysis, RSV infection with co-pathogens, nosocomial infection, higher median band cell percentage and lower median platelet concentration were significantly associated with the need for assisted ventilation (Table 5).

**Table 5:** Characteristics associated with need for assisted ventilation

Characteristic	Assisted ventilation		No ventilation		*P*-value
	No	%	No	%	
Age					
< 6	45	76.3	115	68.9	0.18
≥ 6	14	23.7	52	31.1	
Gender					
Male	32	54.2	85	50.9	0.39
Female	27	45.8	82	49.1	
Pre-existing condition					
Yes	29	25.7	84	74.3	0.88
No	30	26.5	83	73.5	
Weight-for-age z score					
Normal	33	24.6	101	75.4	0.54
Underweight	26	28.3	66	71.7	
HIV status					
Infected	2	50.0	2	50.0	0.28
Uninfected	57	25.7	165	74.3	
Co-pathogen					
Yes	32	54.2	38	22.8	<0.001
No	27	45.8	129	77.2	
RSV subtype					
A	49	83.1	132	79.0	
B	10	16.9	35	21.0	
Pattern of infection					
Nosocomial	10	16.9	12	7.2	0.03
Community-acquired	49	83.1	155	92.8	
Median CRP (mg/L)	19.95		11.8		0.08
Median WCC (x 10^9^/L)	10.9		11.2		0.95
Median percent band cells (%)	10.0		8.0		0.01
Median Hb (g/dl)	11.6		10.9		0.18
Median Platelet count (x 10^9^/L)	382.0		424		0.01

Underweight: Weight-for-age z-score <2; *CRP* C-reactive protein, *WCC* White cell count, *Hb* Haemoglobin

### Factors associated with nosocomial RSV infection

Factors significantly associated with nosocomial infection on univariate analysis included age 6 months or older and pre-existing conditions. However, on multivariate analysis age 6 months or older was the only factor independently associated with nosocomial infection with RSV (Table 6). The odds of nosocomial infection was 3.35 times higher in infants and children aged 6 months or older, compared to younger infants (adjusted OR = 3.35 (1.20–9.36); *p* = 0.02).

**Table 6:** Risk factors for nosocomial RSV infection

Risk factor	Nosocomial *N* = 22 n/n(%)	Crude OR (95 % CI)	*p* value	Adjusted ^b^ OR (95 % CI)	*p* value
Age					
< 6 months	9/160(5.6)	1		1	
≥ 6 months	13/66(19.7)	4.11 (1.66–10.18)	0.002	3.35 (1.20–9.36)	0.02
Gender					
Male	9/117(7.7)	1		1	
Female	13/109(11.9)	1.63 (0.67–3.97)	0.29	1.90 (0.69–5.24)	0.22
Weight-for-age z score					
Appropriate	10/134(7.5)	1		1	
Underweight	12/92(13)	1.86 (0.78–4.51)	0.17	1.55 (0.56–4.3)	0.40
Pre-existing conditions other than HIV infection					
Yes	17/113(15)	1		1	
No	5/113(4.4)	0.23 (0.09–0.74)	0.01	0.40 (0.13–1.27)	0.12
HIV status ^a^					
Unexposed uninfected	15/164(9.2)	1		1	
Exposed uninfected	4/47(8.5)	0.93 (0.29–2.93)	0.89	1.26 (0.36–4.40)	0.72
Exposed infected	1/4(25.0)	3.31 (0.32–33.84)	0.31	4.31 (0.34–55.44)	0.26
RSV genotype					
N A1	14/127(11.0)	1		1	
NA2	0/9(0)	NA		NA	
ON1	5/45(11.1)	1.01 (0.34–2.98)	0.34	0.84 (0.26–2.73)	0.77
BA4	3/45(6.7)	0.58 (0.16–2.11)	0.16	0.70 (0.18–2.81)	0.62

*OR* (*95* % *CI*) Odds ratio (95 % confidence interval)

^a^ HIV status not known in 11; n/n = stratum specific prevalence. NA = Not applicable

^b^ Model adjusted for nutritional status, HIV status, age, sex, genotype and pre-existing conditions

## Discussion

In our study of hospitalised children in Cape Town, the RSV cases were recorded from January until October with most cases occurring in May 2012. This is consistent withdata from the severe acute respiratory infections (SARIs) surveillance programme from South Africa which showed that despite circulation throughout the year, the RSV season in the southern hemisphere peaked between February and May [14].

Infants under 6 months of age constituted 71 % of the cohort, which is consistent with a previous study from Kenyain which 58 % of children with RSV were under 6 months of age [25]. In contrast, a study from Indonesia reported the highest incidence of RSV among children aged 6 to 24 months, with low incidence in infants less than 6 months and no cases detected in those under 3 months [26]. These differences may be partially explained by the population-based design, rather than being hospital based [27, 28]. However, another population-based study carried out in the United States found that the highest rate of hospitalisation was in infants who were less than 1 month old [29]. The full explanation for these age differences remain unclear.

In the study cohort, 50 % of the children had pre-existing conditions other than HIV infection. Notable underlying conditions were prematurity (51 %) and congenital heart disease (30 %). Other important conditions were malnutrition and trisomy 21. Unlike the previous study in South Africa carried out when paediatric HIV exposure and infection prevalence was very high in hospitalised children [6, 12], the present study recorded a lower HIV exposure prevalence of 21 % and infection prevalence of only 2 %. The low HIV infection prevalence is primarily due to reduced perinatal HIV transmission resulting from more widely implemented prevention of mother-to-child interventions in South Africa [30]. Prematurity and other high risk conditions however remain prevalent. Immunoprophylaxis may be used in children with underlying conditions as no vaccines currently exist for RSV. Palivizumab has been found to reduce by half the risk of hospitalisation due to RSV infection in these high-risk infants and children, but the high cost prohibits its use in most low- and middle-income countries, including South Africa [31].

Cough was the commonest symptom which was recorded in 86 % of the children, followed by difficulty in breathing (tight chest) in 51 % and fever in 42 %. Wheeze was recorded in only 9 %. Though there was no comparison of the clinical features between RSV positive and RSV negative children in our study, the symptoms were similar to the findings in a study from Pakistan [32]. In that study, the most frequent clinical findings in RSV cases were cough (99 %) and fever (91 %), though wheezing was also present in 91 % of the cases. In the study, clinical features were not statistically significantly different when comparing RSV positive and negative children presenting with acute respiratory infection [32]. In contrast, a study from Jordan that compared presenting symptoms as reported by parents in RSV-positive and RSV-negative children, showed that RSV-positive subjects were more likely to present with cough and shortness of breath and less likely to present with fever, decreased activity, diarrhoea, or vomiting [33]. The differences recorded in the clinical symptoms of RSV may partly explain the limited usefulness of clinical scores. It is desirable to have a clinical index score for RSV disease in order to reduce the use of antibiotics and unnecessary laboratory investigations as well as to facilitate institution of appropriate infection control measures to reduce nosocomial spread. A clinical score which will take into consideration key bronchiolitis symptoms may be useful especially in settings with limited laboratory support and cost. Bronchiolitis symptoms are however not specific to RSV and unfortunately, lack of uniformity of clinical scores and lack of useful association with outcomes have limited their usefulness. Most of the scoring systems have also not been assessed for reliability and validity [34]. Oxygen saturation in a scoring system would also perform poorly in a study cohort of RSV cases such as ours with a high percentage of congenital heart disease and chronic lung disease. It has been suggested that repeat observations may rather provide a more valid overall assessment as the course of bronchiolitis is variable and dynamic [35].

Co-pathogens were detected in 31 % of the patients with RSV infection, most commonly rhinovirus (26 %), adenovirus (14 %) and para-influenza virus (12 %). It is noteworthy that bacterial co-infectionswere only detected in 29 % of our RSV-positive cases while viral co-infections accounted for 62 %. Unsurprisingly, most of these co-pathogens were recovered from the respiratory tract. Although, it may be difficult to determine the relative contributions of the different pathogens that were identified in causing disease or simply colonising the respiratory tract, the presence of any co-pathogen was associated with ventilation, an indicator of severe respiratory tract infection. A high percentage of the patients received antibiotics which was consistent with WHO recommendations to treat all children with pneumonia with antibiotics, to reduce pneumonia-associated mortality [36].

Ten percent of the children in our cohort had nosocomially-acquired RSV infection, similar to findings reported by Madhi et al. [6]. A Canadian multicentre study reported a lower prevalence of 6 % [37]. Nosocomial prevalence rates of more than 20 % have been documented in studies investigating RSV infection in infant, ICU and haematology-oncology units [7, 38, 39]. In the present study, logistic regression analysis showed that the adjusted odds of developing nosocomial infection was more than three-fold greater in children aged6 months or older, compared to younger infants. It is possible that younger children acquire RSV in the community due to the intense transmission and those older represent a specific group of patients in ourhospital. Hall et al reported that the risk of nosocomial infection could not be related to age or underlying disease but rather to the duration of hospital stay [4]. In the study by Madhi et al, nosocomial infection was found to be greater in children with risk factors which included prematurity, congenital heart disease and chronic lung disease [6]. Differences between studies is likely to continue as a result of variations in design, study location and study population. Nosocomial infections should be anticipated when there is a community outbreak and preparations made to prevent in-patients from acquiring the disease during the course of their hospitalisation. Cheng et al. have advocated for regular surveillance of the shedding of virus and confirmation of viral clearance before discontinuation of infection control measures in order to prevent further outbreaks in other patients [40]. Another important consideration which is often overlooked is the role of staff in the spread of RSV infection. About 40 % of medical personnel in an infant ward in one study were shown to have acquired the virus during a nosocomial outbreak and appeared to play a major role as vectors for transmission in the ward. [35] Staff may also continue to be infected from close contact with ill infants or contaminated secretions [41].

RSV A and B groups co-circulated during the 2012 season, although groupA predominated (80 %). Three group A genotypes (NA1, NA2 and ON1) were detected with NA1 predominating (70 %) whereas only one group B genotype (BA4) was detected. The ON1 genotype though first isolated in Canada in 2011 has since been isolated in other settings, including RCWMCH [42]. The ON1 strain was reported to have spread and nearly replaced other RSV A strains in subsequent epidemic seasons in central Italy and Cyprus [13]. The ON1 genotype already appears to have become diversified as variants distinct from the original viruses were found among children admitted to a rural hospital in Kenya during 2012 [43].

A total of 45 ON1 isolates were reported in the present study. A previous study described the first eight ON1 isolates identified at our hospital [42]. The present study showed that the genotype occurred throughout the RSV season and there was no significant difference in its distribution between nosocomial and community-acquired infection. In contrast to Panayiotou et al who reported that the mildest illness was associated with the novel ON1 genotype compared to infections with genotypes GA2 and BA (RSV B) [27], no differences were recorded in the pattern of infection caused by individual RSV groups or genotypes in our study. As the epidemiologic and pathophysiologic characteristics of RSV suggest that there is potential for a different type of vaccine for different target populations [43], the clinical and molecular epidemiology of RSV strains should be monitored to identify new and emerging genotypes.

As with many retrospective studies, some variables could not be analysed as a result of either incompleteness or absence of data. For example, there was no information on breastfeeding or exposure to cigarette smoke in the patient records, and HIV results were not available for some children. The study was limited by being a single centre, single season and the lack of a control group. The convenience sampling method utilised in this study may have inadvertently selected nosocomial cases. Furthermore, the small number of nosocomial cases did not allow for a more rigorous exploration of associations. As the host and viral factors associated with RSV disease are highly dynamic, adequately powered prospective multi-centre studies are required to address these questions, explore the risk factors associated with nosocomial infection in our setting comprehensively, and unravel more facts about this complex virus infection in our setting.

## Conclusions

RSV lower respiratory tract infection at RCWMCH has been characterised with a large percentage of children having pre-existing conditions. The majority of children presented with cough and difficulty in breathing. Approximately one tenth of the RSV infections were nosocomial with age 6 months or older a risk factor for nosocomial acquisition. Though both RSV groups co-circulated during the season, group A was predominant, and three different A genotypes were documented including the novel ON1 genotype, first reported in Canada in 2011. Continued surveillance of the clinical epidemiology and phylogeny of RSV strains is important for vaccine development.

## References

[1] Nair H, Nokes D, Gessner B (2010). Global burden of acute lower respiratory infections due to respiratory syncytial virus in young children: a systematic review and meta-analysis. Lancet.

[2] Henderson F, Collier A, Cyde WJ (1979). Respiratory syncytial virus infection, re-infections and immunity. N Eng J Med.

[3] Hall CB (1982). Respiratory syncytial virus: its transmission in the hospital environment. Yale J Biol Med.

[4] Hall CB (2000). Nosocomial respiratory syncytial virus infections: the “Cold War” has not ended. Clin Infect Dis.

[5] Langley JM, LeBlanc JC, Wang EE (1997). Nosocomial respiratory syncytial virus infection in Canadian pediatric hospitals: a Pediatric Investigators Collaborative Network on Infections in Canada Study. Pediatrics.

[6] Madhi SA, Ismail K, O’Reilly C (2004). Importance of nosocomial respiratory syncytial virus infections in an African setting. Trop Med Int Health.

[7] Thorburn K (2009). Pre-existing disease is associated with a significantly higher risk of death in severe respiratory syncytial virus infection. Arch Dis Child.

[8] Sullender WM (2000). Respiratory syncytial virus genetic and antigenic diversity. Clin Microbiol Rev.

[9] Hendry RM, Talis AL, Godfrey E (1986). Concurrent circulation of antigenically distinct strains of respiratory syncytial virus during community outbreaks. J Infect Dis.

[10] Venter M, Madhi SA, Tiemessen CT (2001). Genetic diversity and molecular epidemiology of respiratory syncytial virus over four consecutive seasons in South Africa: identification of new subgroup A and B genotypes. J Gen Virol.

[11] Cane PA (2001). Molecular epidemiology of respiratory syncytial virus. Rev Med Virol.

[12] Visser A, Delport S, Venter M (2008). Molecular epidemiological analysis of a nosocomial outbreak of respiratory syncytial virus associated pneumonia in a kangaroo mother care unit in South Africa. J Med Virol.

[13] Pierangeli A, Trotta D, Scagnolari C, et al. Rapid spread of the novel respiratory syncytial virus A ON1 genotype, central Italy, 2011 to 2013. Euro Surveill. 2014;19(26). 10.2807/1560-7917.es2014.19.26.20843 25011065

[14] Pretorius MA, van Niekerk S, Tempia S (2013). Replacement and positive evolution of subtype A and B respiratory syncytial virus G-protein genotypes from 1997-2012 in South Africa. J Infect Dis.

[15] de-Paris F, Beck C, de Souza Nunes L (2014). Evaluation of respiratory syncytial virus group A and B genotypes among nosocomial and community-acquired pediatric infections in Southern Brazil. Virol J.

[16] Amanatidou V, Apostolakis S, Spandidos DS. Genetic diversity of the host and respiratory syncytial virusinduced lower respiratory tract infection, Pediatr Inf Dis J.2009; 28(2):135-40. 10.1097/INF.0b013e31818c8d17 19106772

[17] Walsh EE, McConnochi KM, Long CE (1997). Severity of respiratory syncytial virus infection is related to virus strain. J Infect Dis.

[18] Fodha I, Vabret A, Ghedira L (2007). Respiratory syncytial virus infections in hospitalized infants: association between viral load, virus subgroup, and disease severity. J Med Virol.

[19] Papadopoulos NG, Gourgiotis D, Javadyan A (2004). Does respiratory syncytial virus subtype influences the severity of acute bronchiolitis in hospitalized infants?. Respir Med.

[20] Machado AF, Sallum MA, Vilas Boas LS (2008). Molecular characterization of strains of respiratory syncytial virus identified in a hematopoietic stem cell transplant outpatient unit over 2 years: Community or nosocomial?. Biol Blood Marrow Transplant.

[21] Garcia R, Raad I, Abi-Said D (1997). Nosocomial respiratory syncytial virus infections: prevention and control in bone marrow transplant patients. Infect Control Hosp Epidemiol.

[22] High-dependency unit. A dictionary of Nursing. 2008. Encyclopedia.com. Available at: www.encylopedia.com. Accessed 11 Jul 2015.

[23] World Health Organization Pneumonia Vaccine Trial Investigators’ Group: Standardization of interpretation of chest radiographs for the diagnosis of pneumonia in children. 2001. http://aps.who.int/iris/bitstream/10665/66956/1/WHO_V_and_B_01.35.pdf. Accessed 26 Sept 2013.

[24] Peret T, Hall CB, Schnabel KC (1998). Circulation patterns of genetically distinct group A and B strains of human respiratory syncytial virus in a community. J Gen Virol.

[25] Openshaw PJ, Tregoning JS (2005). Immune responses and disease enhancement during respiratory syncytial virus infection. Clin Microbiol Rev.

[26] Simoes E, Mutyara K, Soh S (2011). The epidemiology of respiratory syncytial virus lower respiratory tract infections in children less than 5 years of age in Indonesia. Pediatr Infect Dis J.

[27] Panayiotou C, Richter J, Koliou M (2014). Epidemiology of respiratory syncytial virus in children in Cyprus during three consecutive winter seasons (2010–2013): Age distribution, seasonality and association between prevalent genotypes and disease severity. Epidemiol Infect.

[28] Zhang X, Liu L, Qian L (2014). Clinical characteristics and risk factors of severe respiratory syncitial virus-associated acute lower respiratory tract infections in hospitalized infants. World J Pediatr.

[29] Hall CB, Weinberg GA, Blumkin AK (2013). Respiratory syncytial virus-associated hospitalizations among children less than 24 months of age. Pediatrics.

[30] Barron P, Pillay Y, Doherty T (2013). Eliminating mother-to-child HIV transmission in South Africa. Bull World Health Organ.

[31] Andabaka T, Nickerson JW, Rojas-Reyes M, Rueda J, Bacic Vrca V, Barsic B. Monoclonal antibody for reducing the risk of respiratory syncytial virus infection in children. Cochrane Database of Systematic Reviews 2013; doi:10.1002/14651858. 10.1002/14651858.CD006602.pub4 23633336

[32] Aamir UB, Alam MM, Sadia H, et al. Molecular Characterization of Circulating Respiratory Syncytial Virus (RSV) Genotypes in Gilgit Baltistan Province of Pakistan during 2011-2012 Winter Season. PLoS ONE 2013; doi:10.1371/journal.pone.0074018 10.1371/journal.pone.0074018 PMC3772930 24058513

[33] Halasa N, Williams J, Faouri S, et al. Natural history and epidemiology of respiratory syncytial virus infection in the Middle East: Hospital surveillance for children under age two in Jordan.Vaccine. 2015; doi:10.1016/j.vaccine.2015.08.048. 10.1016/j.vaccine.2015.08.048 PMC7115487 26314623

[34] Bekhof J, Reimink R, Brand PL (2014). Systematic review: insufficient validation of clinical scores for the assessment of acute dyspnoea in wheezing children. Paediatr Respir Rev.

[35] Subcommittee on Diagnosis and Management of Bronchiolitis. Diagnosis and Management of Bronchiolitis. Pediatrics. 2006; doi:10.1542/peds.2006-2223.

[36] World Health Organization. Revised WHO classification and treatment of childhood pneumonia at health facilities – Evidence summaries. http://apps.who.int/iris/bitstream/10665/137319/1/9789241507813_eng.pdf. Accessed 22 July 2015 25535631

[37] Wang EE, Law BJ, Stephens D (1995). Pediatric Investigators Collaborative Network on Infections in Canada (PICNIC) prospective study of risk factors and outcomes in patients hospitalized with respiratory syncytial viral lower respiratory tract infection. J Pediatr.

[38] Hall C, Douglas RJ, Geiman J (1975). Nosocomial respiratory syncytial virus infection. N Engl J Med.

[39] Gels S, Prifert C, Weissbrich B (2013). Molecular characterization of a respiratory syncytial virus outbreak in a hematology unit in Heideberg, Germany. J Clin Microbiol.

[40] Cheng FW, Lee V, Shing MM (2008). Prolonged shedding of respiratory syncytial virus in immunocompromised children: implication for hospital infection control. J Hosp Infect.

[41] Hall CB, Geiman JM, Douglas RG (1978). Control of nosocomial respiratory syncytial viral infections. Pediatrics.

[42] Valley-Omar Z, Muloiwa R, Hu N (2013). Novel Respiratory Syncytial Virus subtype ON1 among children, Cape Town, South Africa, 2012. Emerg Infect Dis.

[43] Eshaghi A, Duvvuri VR, Lai R, et al. Genetic variability of human respiratory syncytial virus A strains circulating in Ontario: a novel genotype with a 72 nucleotide G gene duplication. PLoS One. 2012; doi:10.1371/journal.pone.0032807. 10.1371/journal.pone.0032807 PMC3314658 22470426

